# Coronary artery bypass grafting on the beating heart: 13-years experience in a single center

**DOI:** 10.1186/1749-8090-8-S1-O172

**Published:** 2013-09-11

**Authors:** G Bogáts, Z Hegedüs, M Bitay, L Csepregi, A Szabó-Biczók, S Varga, G Iglói, G Bari

**Affiliations:** 1 Department of Cardiac Surgery, University of Szeged, Szeged, Hungary

## Background

The relative value of off-pump coronary bypass (OPCAB) surgery as compared to conventional coronary artery bypass operation is still debated. We started a prospective study in 2000 with the aim of using exclusively OPCAB for surgical myocardial revascularization in a single center. The present report analyzes the 13-years results of this approach in a large consecutive patient population.

## Methods

Of the 6111 coronary bypass operation performed in our center between January 2000 and December 2012, 5472 were carried out off-pump. Since 2004 OPCAB surgery was performed in more than 99% of cases, conventional CABG was reserved for patients in cardiogenic shock. The OPCAB approach consisted of the use of a single pericardial sling, pressure stabilizing device, aortocoronary shunt and elastic snares. Prophylactic intra-aortic balloon pump counter-pulsation was used in 495 (9%) of cases.

## Results

The OPCAB hospital mortality was 1.5% (85/5472). Perioperative myocardial infarction occurred in 60 (1.1%), stroke in 85 (1.2%), major adverse cardiac event in 132 (2.4%) cases. 121 patients needed reoperation for bleeding (2.2%). The mean length of hospital stay was 9.9 (SD 8.5) days.

## Conclusions

The shift towards beating-heart surgery provides acceptable results, but does not eliminate perioperative mortality and morbidity.

